# Red propolis hydroalcoholic extract inhibits the formation of *Candida albicans* biofilms on denture surface

**DOI:** 10.4317/jced.56843

**Published:** 2020-07-01

**Authors:** Karla-Lorene-de França Leite, Mariana-Leonel Martins, Mariana-Marinho-Davino de Medeiros, Natanael-Victor-Furtunato Bezerra, Camila-Santos-de Mattos Brito, Leopoldina-de Fátima-Dantas de Almeida, Yuri-Wanderley Cavalcanti

**Affiliations:** 1 Department of Pediatric Dentistry and Orthodontics, School of Dentistry, Federal University of Rio de Janeiro, Rio de Janeiro, RJ, Brazil; 2 Department of Prosthodontics and Periodontology, Piracicaba Dental School, University of Campinas, Piracicaba, SP, Brazil; 3 Department of Clinical and Social Odontology, Federal University of Paraíba, João Pessoa, PB, Brazil

## Abstract

**Background:**

To evaluate the antifungal activity of the red propolis hydroalcoholic extract (RPHE) against *Candida albicans* biofilms.

**Material and Methods:**

The minimum inhibitory and fungicidal concentrations (MIC and MFC) of the RPHE were determined by the microdilution technique. *C. albicans* biofilms were formed on the surface of resin specimens preconditioned with artificial saliva (1h). The specimens (N=48) were equally divided according to the four solutions used for anti-biofilm evaluation (n=12 per group). After overnight incubation, biofilms were daily exposed (2x/day for 15 min) along 3 days with 3% RPHE, 0.12% chlorhexidine (CHX), 50,000 IU/mL nystatin (NYS) or saline (0.9% NaCl). Biofilms were analyzed regarding the number of viable microorganisms (CFU/mL), the metabolic activity (MTT assay) and the proportion of hyphae (optical microscopy).

**Results:**

The MIC and MFC of RPHE were respectively 0.29 mg/mL (0.03%) and 1.17 mg/mL (0.12%). There was no difference in the microorganisms’ viability (CFU/mL) among groups treated with RPHE (4.92×103), CHX (3.33×102) or NYS (6.8×104), being all of them different from NaCl (3.93×107) (*p*<0.05). The CHX (0.133) had the lowest metabolic activity (*p*<0.05), followed by RPHE (0.292) and NYS (0.302) (*p*>0.05). All experimental groups had a mean proportion of hyphae <10%, lower than NaCl (70%).

**Conclusions:**

RPHE has antifungal activity against *C. albicans* biofilms, suggesting its use for the biofilm control on denture surfaces.

** Key words:**Propolis, Candida albicans, biofilm, dentures, antifungal agents.

## Introduction

Oral candidiasis is an opportunistic infection related to the excessive proliferation of *Candida*, as a result of the homeostatic imbalance of oral microbiota (1). In many cases, *Candida* infection is associated with the use of removable dentures, especially among denture wearers who are not able to maintain satisfactory hygiene of their prosthesis (2,3).

Denture cleaning solutions are frequently recommended for the treatment of such infections, associated or not with the use of local antifungals (3,4). The efficacy of antifungal agents has been proven by a systematic review of the literature (5). Although the efficacy of denture cleansing solutions is also proven in the literature, new alternatives remain in constant development (4). Moreover, the preventive effect of these solutions has been little explored in the literature (4).

In general, recurrence of denture stomatitis may occur due to discontinuation of antifungal therapy and microbial resistance (6,7). Methods for cleaning prosthetic surfaces may reduce the accumulation of biofilm, but do not prevent recolonization (8). In addition, they may compromise the longevity of dentures by promoting surface changes such as abrasion and increased roughness (9).

Natural products have promoted the search for therapeutic possibilities that are biocompatible and accessible to the population in order to overcome these limitations (10,11). Red propolis, mainly formed by the botanical compounds extraction of *Dalbergia ecastophyllum* from *Apis mellifera* bees, presents phenolic compounds, especially flavonoids. These phytochemicals are associated with antifungal activity (12) and make this product a promising natural antimicrobial agent (13-15).

Red propolis extract has been shown to control the imbalance of oral microbiota homeostasis by reducing the proliferation of Candida (16), as well as being a biocompatible substance compared to synthetic antimicrobials (17,18), which may have undesirable effects, such as microbial resistance (19).

Thus, there is a need to evaluate its antifungal activity, with the purpose of presenting an alternative for the treatment of fungal infections. Therefore, the aim of this study was to evaluate the antifungal activity of the red propolis hydroalcoholic extract (RPHE) against *C. albicans* biofilms developed on the surface of denture base material.

## Material and Methods

-Ethics information

This study did not involve human participants or material collected from human (i.e.: saliva). Biofilms were developed in the presence of artificial saliva. Microorganisms samples used in this study were derived from standardized strains cultured in laboratory.

-Red Propolis Hydroalcoholic Extract

The red propolis hydroalcoholic extract (RPHE) was obtained in the concentration of 30%, provided by Apiário Edimel located in the town of João Pessoa, Brazil (Latitude 7° 3′ 25,756′’; Longitude O 34° 50′ 55,155′’). Red propolis was collected within the Brazilian spring period (23º - 30º) (18). The RPHE was used in its commercial presentation (30% w/v, or 300 mg/mL) for the determination of Minimum Inhibitory Concentration (MIC). For anti-biofilm assays, the RPHE concentration was based on MIC and on a 10× dilution of its commercial form.

-Standardization of Inoculum

*Candida albicans* (ATCC 90028) was reactivated in Petri dishes (AlamarTM, Diadema, BRA) containing Sabouraud Dextrose agar (Himedia LaboratoriesTM, Mumbai, India) and incubated at 37 °C for 48 hours. The inoculum was standardized in RPMI 1640 (Aldrich Chemical Co., St. Louis, USA) using a spectrophotometer (Lgi ScientificTM, São Paulo, BRA). An optical density of 0.1, under 600 nm wavelength, was set to obtain the concentration of 5×106 CFU/mL (20). The final concentration of the inoculum for MIC and biofilm assays were 5×103 and 1×105 CFU/mL, respectively.

-Antifungal Activity

The antifungal activity was determined by means of the minimum inhibitory concentration (MIC), in which a microdilution assay was carried out in a 96-well microtiter plate (AlamarTM, Diadema, São Paulo, BRA), according to M27A3 from CLSI (20). RPHE (30% w/v) and 0.12% chlorhexidine (used as control) were diluted and then 100 μL of the inoculum was inserted into all wells, except in the line corresponding to the sterility control. Final concentrations ranged from 75 to 0.0366 mg/mL for RPHE and from 0.3 to 0.000146 mg/mL for CHX.

After the incubation period in aerobiosis (24 h at 37 ºC), the MIC corresponded to the last dilution of the RPHE in which the presence of microbial precipitate or turbidity in the culture medium after the incubation period was not verified. The determination of MIC was confirmed by the use of 3 mM Resazurin (Sigma, ST. Louis, MO, USA). Resazurin is a colorimetric indicator of cellular respiration, which when in blue represents a state of oxidation and becomes pink when reduced by viable cells detection without the use of a spectrophotometer (21). After incubation for 4 h, the results were visualized by the visual method.

The minimum fungicidal concentration (MFC) was obtained by seeding of 50 μL aliquots of dilutions equal to or greater than MIC. Then, Sabouraud Dextrose plates incubated with MIC aliquots were incubated at 37 °C for 24h. The MFC was considered the lowest concentration of the substance that prevented the visible growth of the subculture, or the formation of up to three Colony Forming Units (CFU) (22). The tests for determination of MIC and MFC were performed in triplicate.

-Anti-biofilm Activity

Anti-biofilm activity was evaluated after developing biofilms on the surface of denture base material. Resin specimens made of polymethylmethacrylate (PMMA) (QC-20, Dentsply Int IncTM, Weybridge, UK) were obtained from prefabricated stainless-steel molds of circular shape (10 mm diameter, 2 mm thickness). The resin was handled according to the manufacturer’s recommendations and inserted from the stainless-steel molds, using a pressure of 1000 N. Polymerization was by immersion in boiling water for 20 min, followed by slow cooling. The specimens were kept in distilled water to release residual monomer for 48h. The selection of specimens occurred after polishing with silicon carbide sandpaper in #400 and #600 granulation. Specimens that presented blisters or roughness were discarded. The specimens (N=48) were autoclaved (121°C, 15 min) immersed in distilled water solution. Then, they were equally divided according to the four solutions used for anti-biofilm evaluation (n=12 per group).

Prior to biofilm formation, the specimens were submitted to the formation of artificial salivary pellicle (23), with 1% carboxymethyl, 0.0084% sodium chloride, 0.12% potassium chloride, 0.0342% phosphate monobasic potassium, 0.0146% calcium chloride, and 0.0052% magnesium chloride. Specimens were then incubated at 37°C for 2 h, in 24-well polystyrene culture plates (model K12-024, KasviTM, São José do Pinhais, BRA). After saliva removal, 2 mL of the *C. albicans* inoculum was inserted. The specimens were then incubated in the presence of the inoculum at 37 °C for 24 h.

After this period, the specimens were individually submitted to treatment (specimen’s immersion, 2 mL) with one of the antimicrobial solutions evaluated in this study: 3% RPHE (based on MIC and on a 10× dilution of RPHE’s commercial presentation), 0.12% Chlorhexidine (CHX), 50,000 IU/mL Nystatin (NYS) and 0.9% Sodium Chloride (NaCl). The treatments were performed twice a day (8:00 am and 4:00 pm) for 3 days, during 15 min. After exposure to the solutions, the specimens were washed in saline solution (2×) and the culture medium was renewed.

After 72 h, specimens were individually transferred to microtubes containing 1 mL saline. Specimens were then vortexed during 1 min for biofilm disruption. Aliquots of suspended cells were used for quantification of viable microorganisms, hyphae quantification and assessing the metabolic activity.

For quantification of number of viable microorganisms, the suspension of cells were serially diluted (10-1 to 10-6) and 10 μl aliquots were seeded (triplicate) on Sabouraud Dextrose agar and incubated at 37 °C for 48 h. After this period, the count of colony forming units (CFU/mL) was verified.

The suspension of cells was also analyzed under an optical microscope using a 100× objective (Carl ZeissTM, Oberkochen, DEU), in order to determine the proportion of hyphae. For this purpose, each slide containing biofilm precipitate was analyzed in at least three different points.

Metabolic activity was assessed by the soluble MTT assay 3-[(4,5-dimethylthiazol-2-yl) -2,5-diphenyltetrazolium bromide] as described by Loures and Levitz (2015) (24), with modifications. The suspended cells from the biofilms were centrifuged for 5 min (CientecTM, Belo Horizonte, BRA). After that, the supernatant was eliminated and 500 μL of MTT solution was added to microtubes, followed by incubation (37 °C, 4 h), without exposure to light. The microtubes were then centrifuged again (5 min) to remove the supernatant. After that, the precipitated was treated with acid alcohol (350 μL) and absorbance was read in a spectrophotometer (490 nm).

-Data Analysis

The results of MIC, MFC and hyphae ratio were tabulated using Microsoft Office Excel 2007TM and analyzed descriptively. SPSS software version 20.0 (IBMTM, Chicago, USA) was used considering a 5% significance level. The non-normal distribution was verified by the Shapiro-Wilk test. The Kruskal-Wallis and Mann-Whitney tests were used to evaluate the number of viable microorganisms and metabolic activity.

## Results

The MIC and MFC of the RPHE were respectively 0.29 mg/mL (0.03%) and 1.17 mg/mL (0.12%). The CHX 0.12% showed fungicidal action in all concentrations tested. Sterility and growth controls indicated, respectively, absence of culture medium contamination and viability of the strains tested.

**Figure 1:**
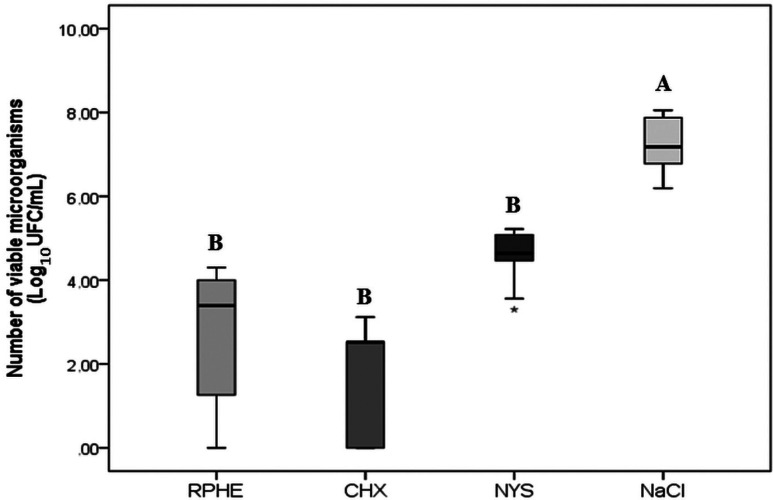
Fungal viability (Log10UFC/mL) in biofilms after treatments. Different letters indicate statistically significant differences between the groups (*p*<0.05, Kruskal-Wallis and Mann-Whitney).

**Figure 2:**
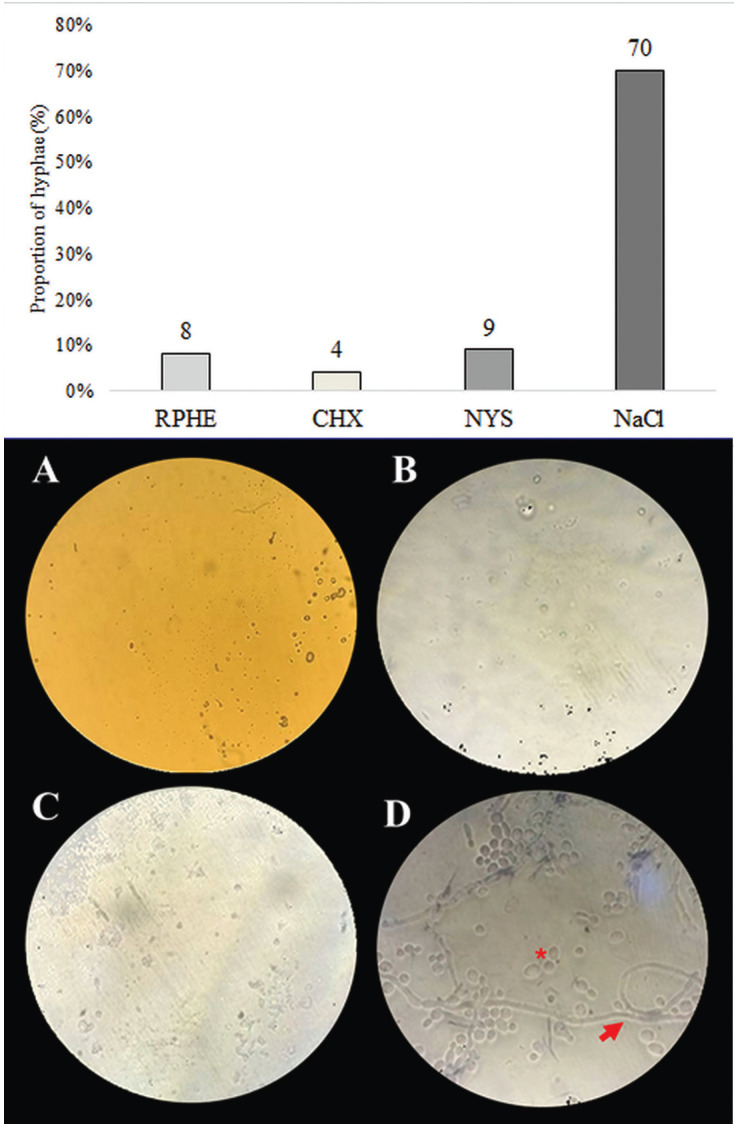
Proportion of hyphae in relation to the quantitative of yeasts in biofilms after treatments. Photomicrography of *C. albicans* biofilms after the end of the experimental protocol to the test products, at 1000× magnification. A = RPHE, B = CHX, C = NYS and D = NaCl. The asterisk (*) represents yeast structures and the arrow represents filamentous structures (hyphae).

There was no statistically significant difference (*p*>0.05) for the number of viable cells among RPHE (4.92×103 CFU/mL), CHX (3.33×102 CFU/mL) and NYS (6,8×104 CFU/mL), although those differed from control (3.93×107 CFU/mL) (*p*<0.05) (Fig. 1). Control group biofilm (NaCl) presented 70% hyphae, whilst the groups exposed to RPHE, CHX and NYS presented less than 10% (Fig. 2).

**Figure 3:**
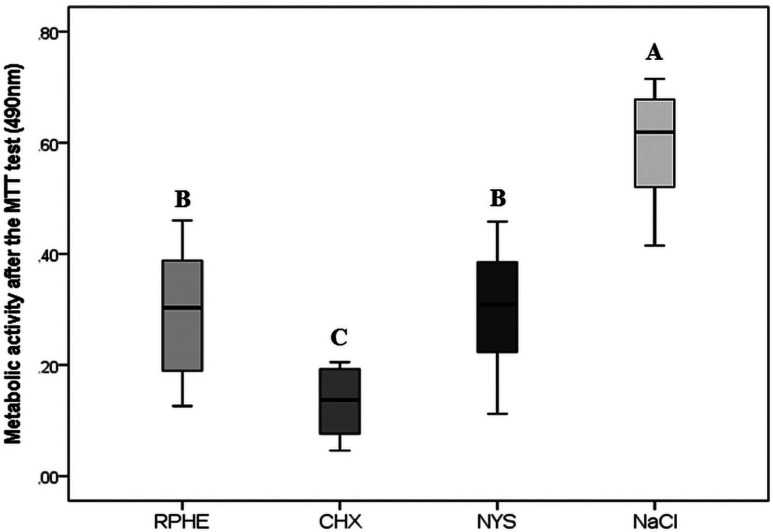
Metabolic activity after the MTT test. Different letters indicate statistically significant differences between the groups (*p*<0.05, Kruskal-Wallis and Mann-Whitney).

Compared to control (100%), the lowest metabolic activity was observed in the group exposed to CHX (22.23%) (*p*<0.05). RPHE (48.82%) and NYS (50.43%) also reduced the metabolic activity and differed significantly from control and CHX (*p*<0.05) (Fig. 3).

## Discussion

The present study demonstrated that the red propolis hydroalcoholic extract inhibited the activity of *C. albicans*, in its planktonic and biofilm form. The RPHE activity was considered satisfactory (0.29 mg/mL), since excellent MIC values for natural products were found below 0.1 mg/mL (25). A previous study confirmed the antimicrobial activity of other red propolis extract against Candida resistant to fluconazole (19), suggesting that the propolis extract could be a promising antimicrobial agent. Results of present study corroborate with this previous finding (19) and added more evidence about the therapeutic potential of a RPHE against *Candida* infections. According to our data, the RPHE reduced the proliferation of viable microorganisms and also decreased the presence of hyphae within the *Candida* biofilm.

The RPHE evaluated in the present study is commercially available from the apiary at a concentration of 30%. The anti-biofilm assays were performed at 3% concentration, corresponding to MIC×100 and to 10× dilution of its commercial presentation. The increased concentration of RPHE (compared to MIC) is due to the robust structure of biofilms, which may influence the resistance compared to planktonic cells (26).

Although there is no consensus attesting to the efficacy of natural products in the treatment of fungal infections (27), indiscriminate use may result in changes that affect application safety (28). On the other hand, the RPHE can be considered promising since the results found for the metabolic activity were similar to nystatin. In addition, the same RPHE (3%) was evaluated in another study that considered accepTable its use in cytotoxicity in cells of fibroblasts (L-929), as their use for 1 minute was able to maintain 43% viable cells (18).

The low cytotoxicity demonstrated by RPHE does not impair its recommendation as a mouthwash or denture cleanser (18). The biofilm model in this study validates its effect for decontamination of dental prostheses, without contact with the oral mucosa. It is still necessary to carry out new studies using different concentrations of the product and with a longer exposure time to evaluate the effect on the color change on the surfaces of dental prostheses (29).

Red propolis and nystatin reduced the metabolic activity at controlled levels of cell proliferation, indicating that those substances have potential for biofilm inhibition. Possibly this similar action between RPHE and nystatin is due to the lower concentration used in the present study, since at 100,000 IU nystatin can cause an increase in roughness and loss of resin hardness when used in up to 15 days (30). The biofilm control through inhibition of cell proliferation is considered effective against oral infections (4,6). All treatment substances evaluated in the present study reduced the number of hyphae present in this biofilm, therefore affecting the complexity and virulence of this microbial community (7).

This *in vitro* study suggests that red propolis extract should be further investigated so that it can be used on disinfection of removable dentures. The number of specimens per group in this study is in accordance with previous investigations (8,18). However, larger sample size is strongly recommended for confirming results. In addition, treatment duration is an aspect that should be improved in future investigations. The number of treatment expositions per day, the duration of expositions and the number of days under treatment can be modified in order to simulate clinical use of the RPHE as a denture cleanser.

Therefore, this study stimulates further investigations that could validate the clinical use of RPHE. Conventional cleansing agents can influence the microbial resistance and promote surface alterations, such as abrasion and the roughness, that decrease the longevity of dentures (1,9). Although we did not evaluate the effects of these treatments on the mechanical properties of PMMA resin, we observed that RPHE could be indicated as an effective cleansing agent for the control of *Candida* biofilms.

## Conclusions

The red propolis hydroalcoholic extract has antifungal activity against *C. albicans* biofilms, suggesting its use for the control of denture biofilms.
